# Supplementary material for: The stability of the primed pool of synaptic vesicles and the clamping of spontaneous neurotransmitter release rely on the integrity of the C-terminal half of the SNARE domain of syntaxin-1A
Source: eLife. 2024 Mar 21;12:RP90775. doi: 10.7554/eLife.90775 (PMC10957171; doi:10.7554/eLife.90775)
Supplement: Figure 1—figure supplement 1—source data 2. [file elife-90775-fig1-figsupp1-data2.zip › Figure 1- Figure Supplement-Data Source 2/Figure 1- Figure Supplement-1A- Data Source 2.pdf]

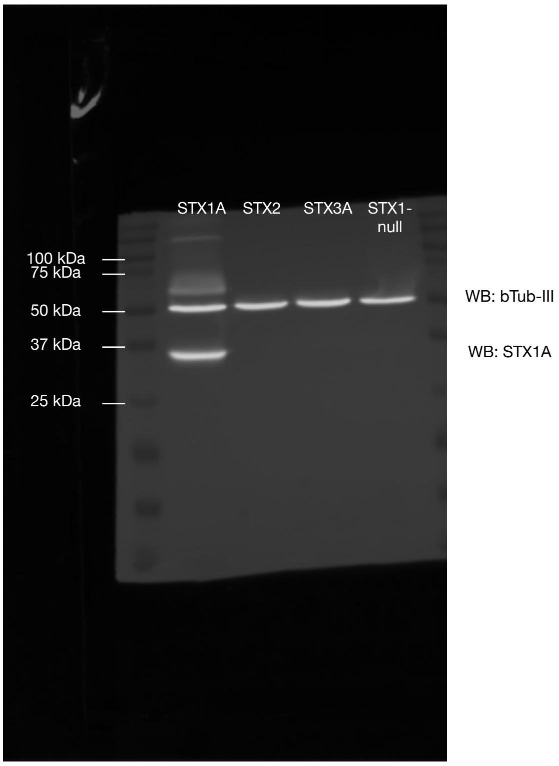

Neuronal lysates, blot **Figure 1 - figure supplement 1A (top)**.
Superposition of detected protein and the image of the marker, Blot's opacity was reduced 30% for the visualization of the marker

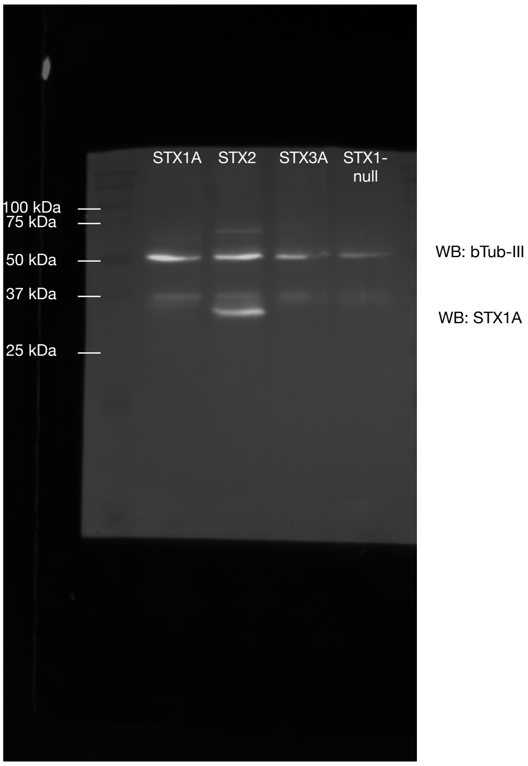

Neuronal lysates, blot **Figure 1 - figure supplement 1A (bottom)**.
Superposition of detected protein and the image of the marker, Blot's opacity was reduced 30% for the visualization of the marker
