# Supplementary figures and images for: The stability of the primed pool of synaptic vesicles and the clamping of spontaneous neurotransmitter release rely on the integrity of the C-terminal half of the SNARE domain of syntaxin-1A
Source: eLife. 2024 Mar 21;12:RP90775. doi: 10.7554/eLife.90775 (PMC10957171; doi:10.7554/eLife.90775)

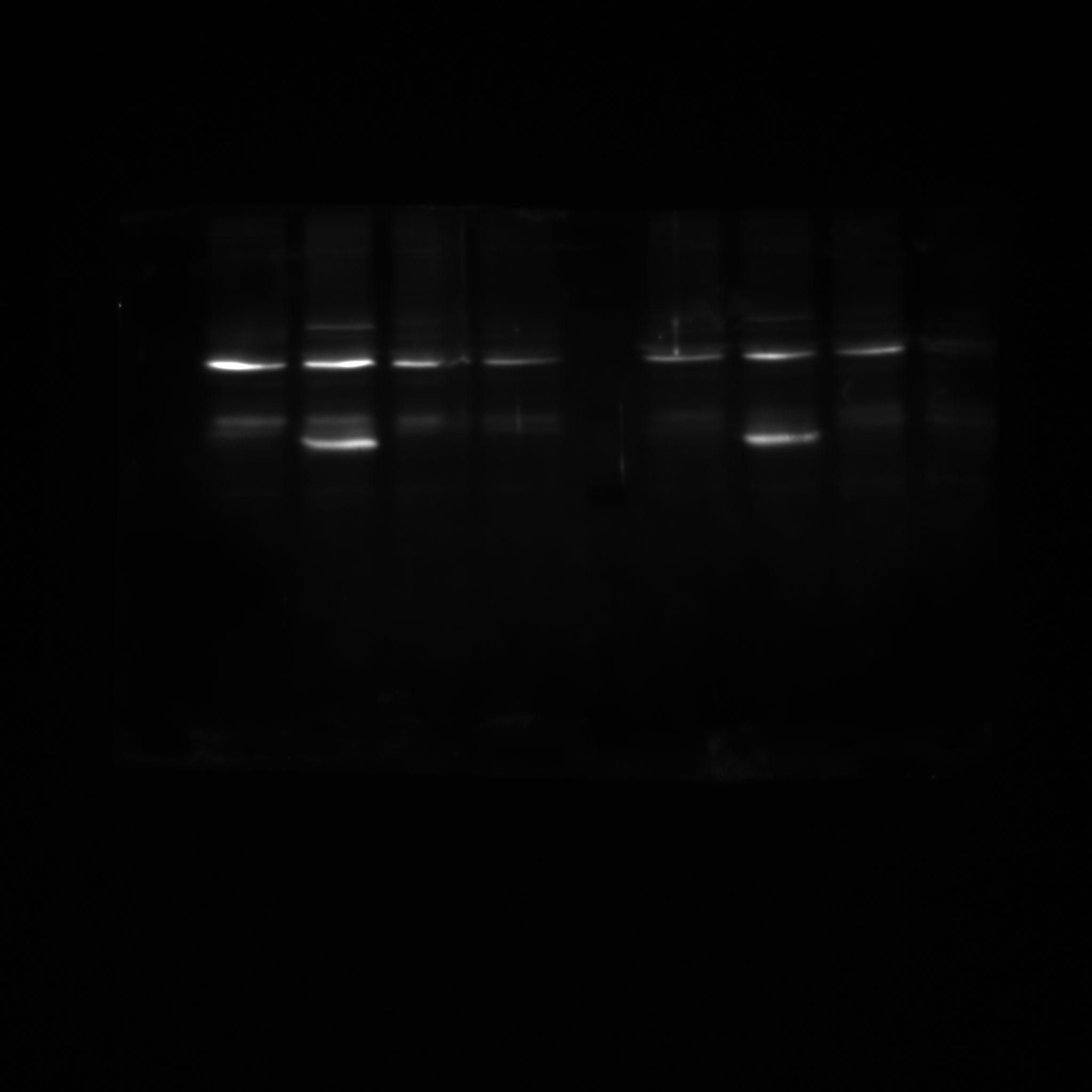

Supplement: Figure 1—figure supplement 1—source data 2. [file elife-90775-fig1-figsupp1-data2.zip › Figure 1- Figure Supplement-Data Source 2/Figure 1 - figure supplement 1A_bottom_whole_blot.Tif]

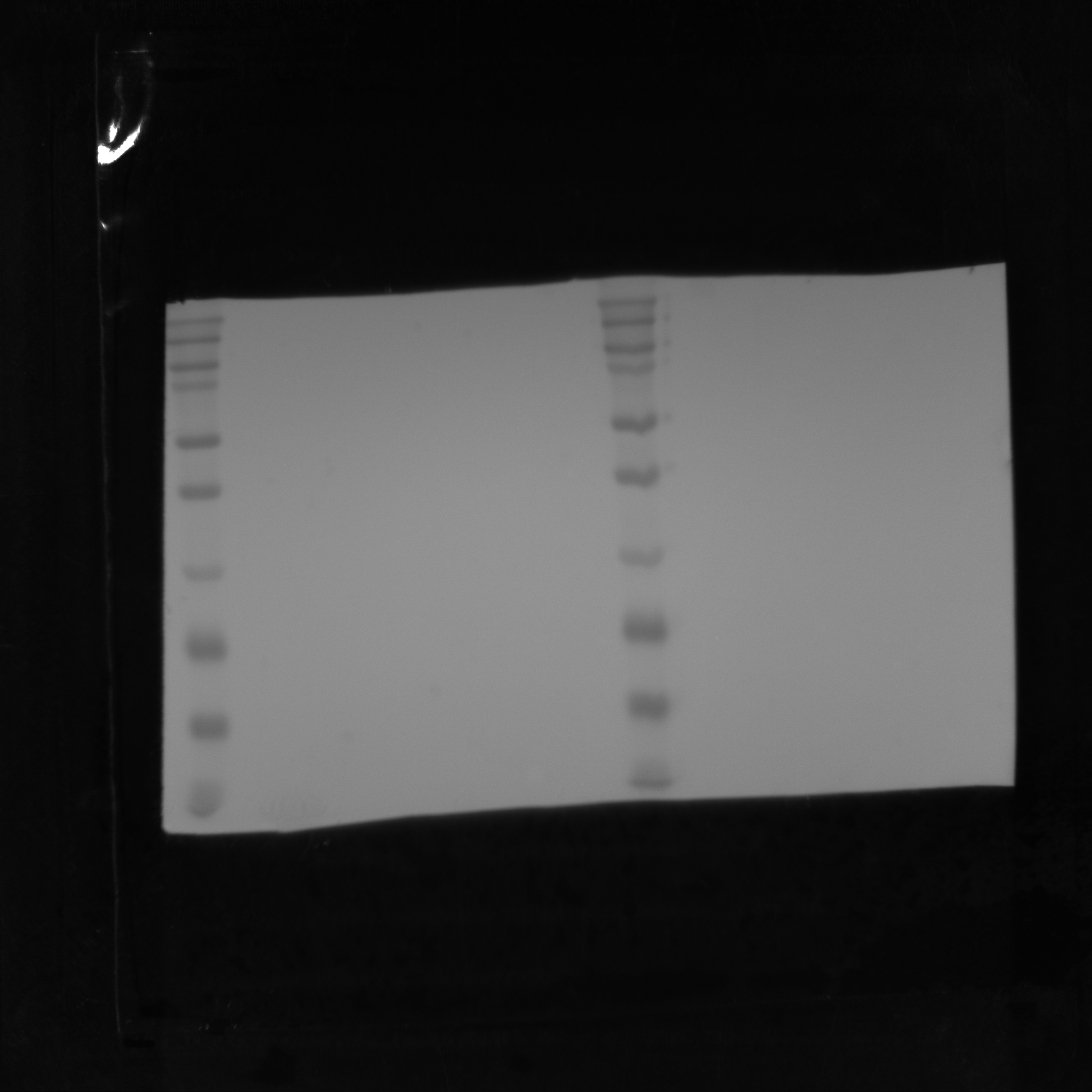

Supplement: Figure 1—figure supplement 1—source data 2. [file elife-90775-fig1-figsupp1-data2.zip › Figure 1- Figure Supplement-Data Source 2/Figure 1-figure supplement_1A_top_maker_precisionkaleidosocope.Tif]

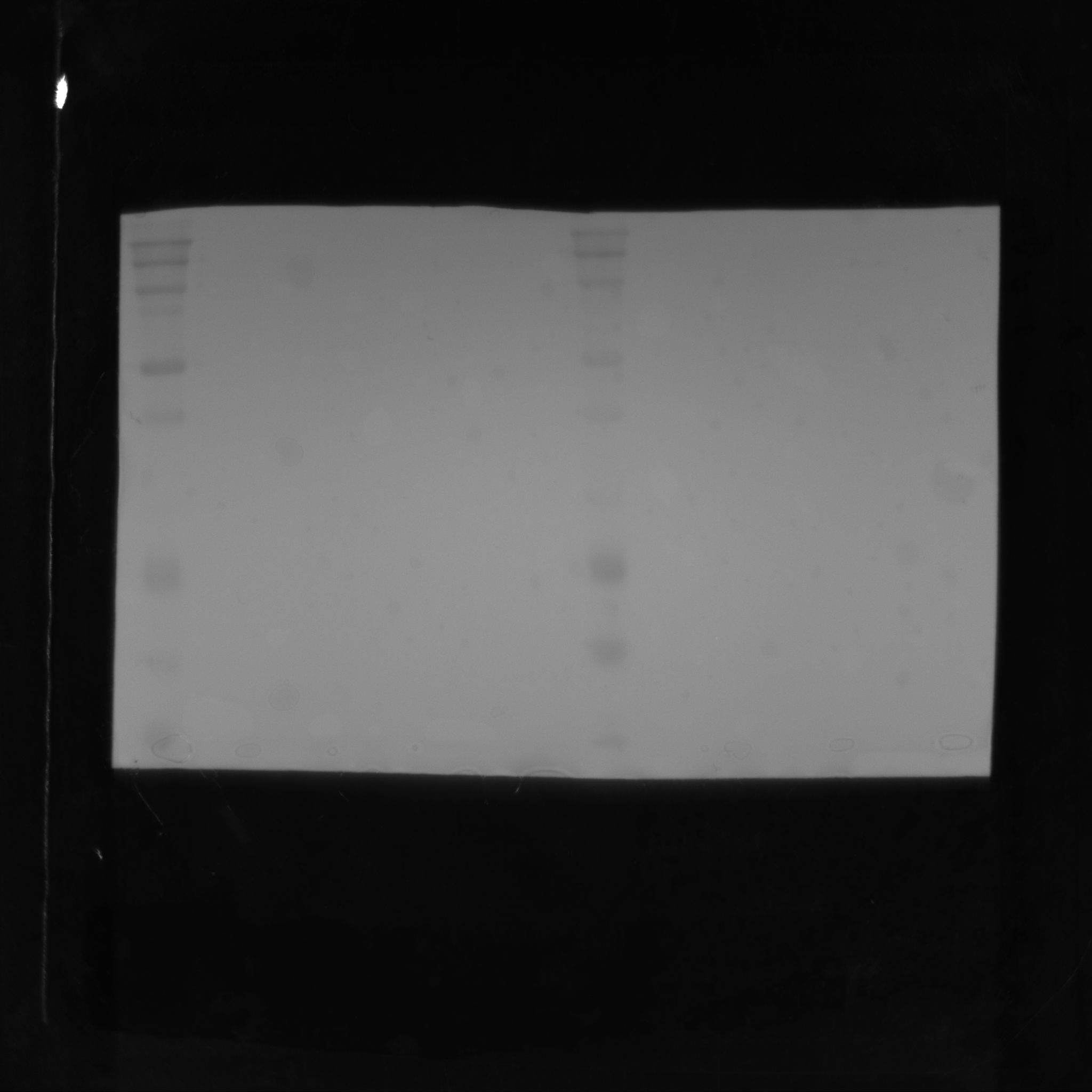

Supplement: Figure 1—figure supplement 1—source data 2. [file elife-90775-fig1-figsupp1-data2.zip › Figure 1- Figure Supplement-Data Source 2/Figure 1-figure_supplement_1A__bottom_marker_precisionkaleidoscope.Tif]

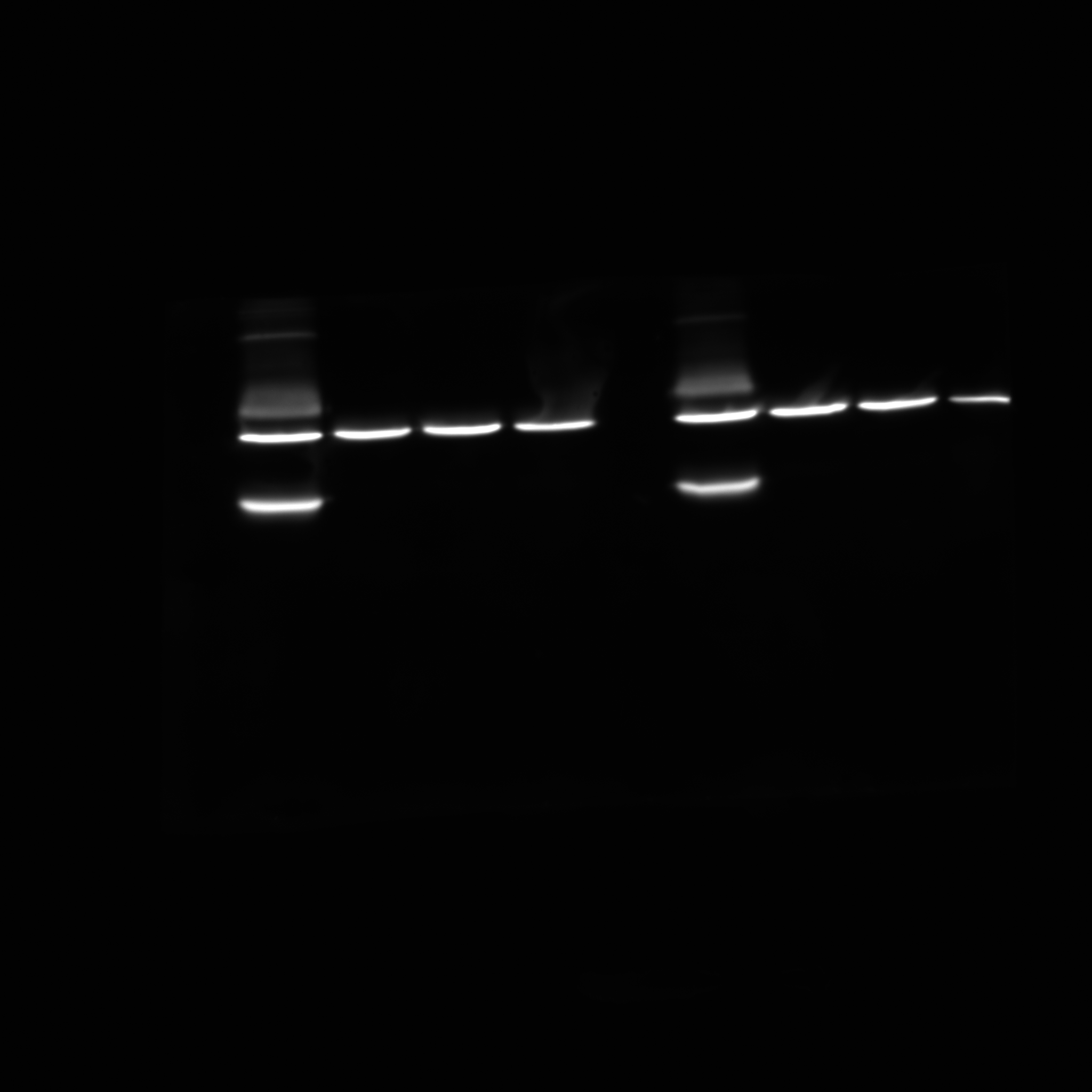

Supplement: Figure 1—figure supplement 1—source data 2. [file elife-90775-fig1-figsupp1-data2.zip › Figure 1- Figure Supplement-Data Source 2/Figure 1-figure_supplement_1A_top_whole_blot.Tif]

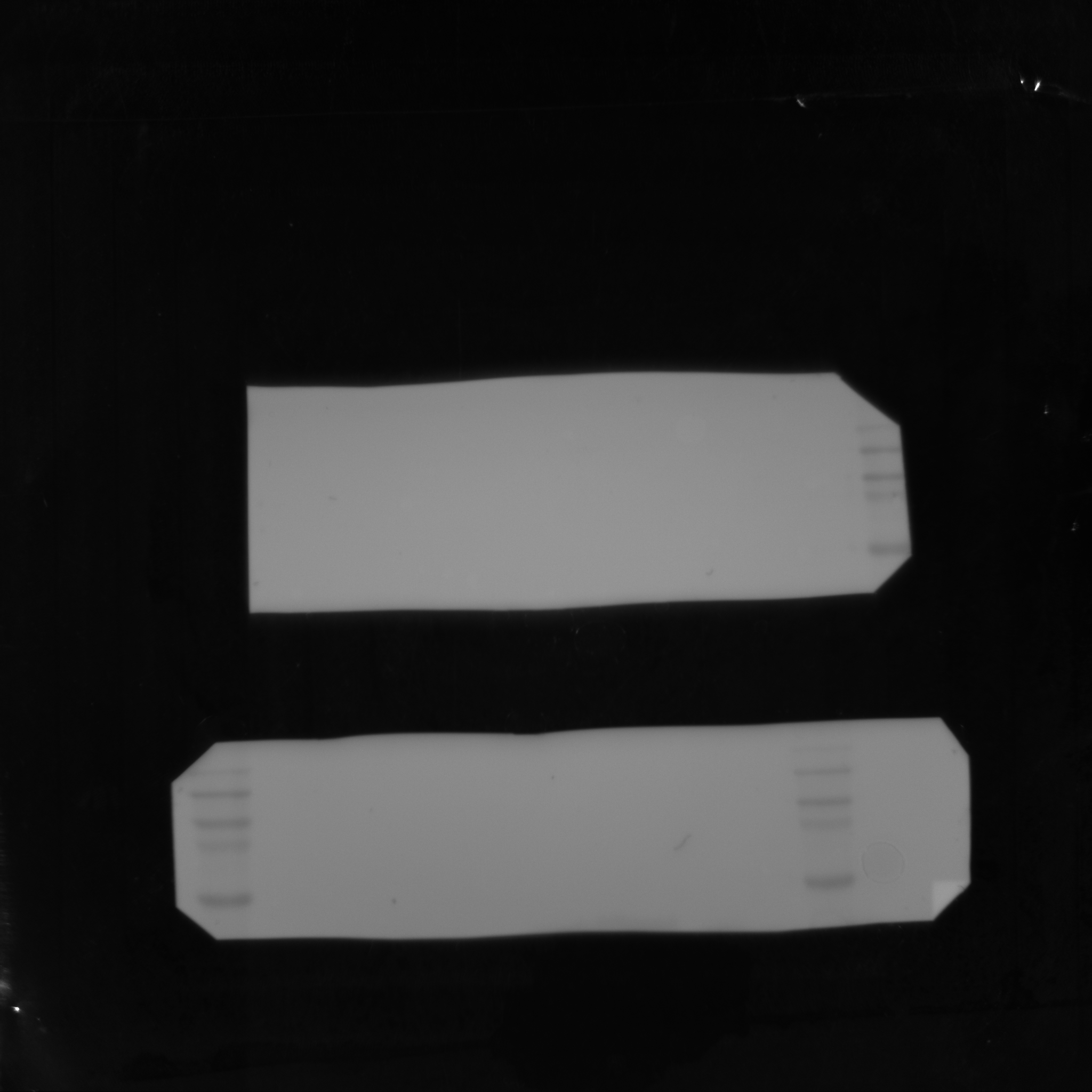

Supplement: Figure 4—source data 2. [file elife-90775-fig4-data2.zip › Figure 4-Data Source 2/Figure 4D_bottom_marker_precisionplusKaleidoscope.Tif]

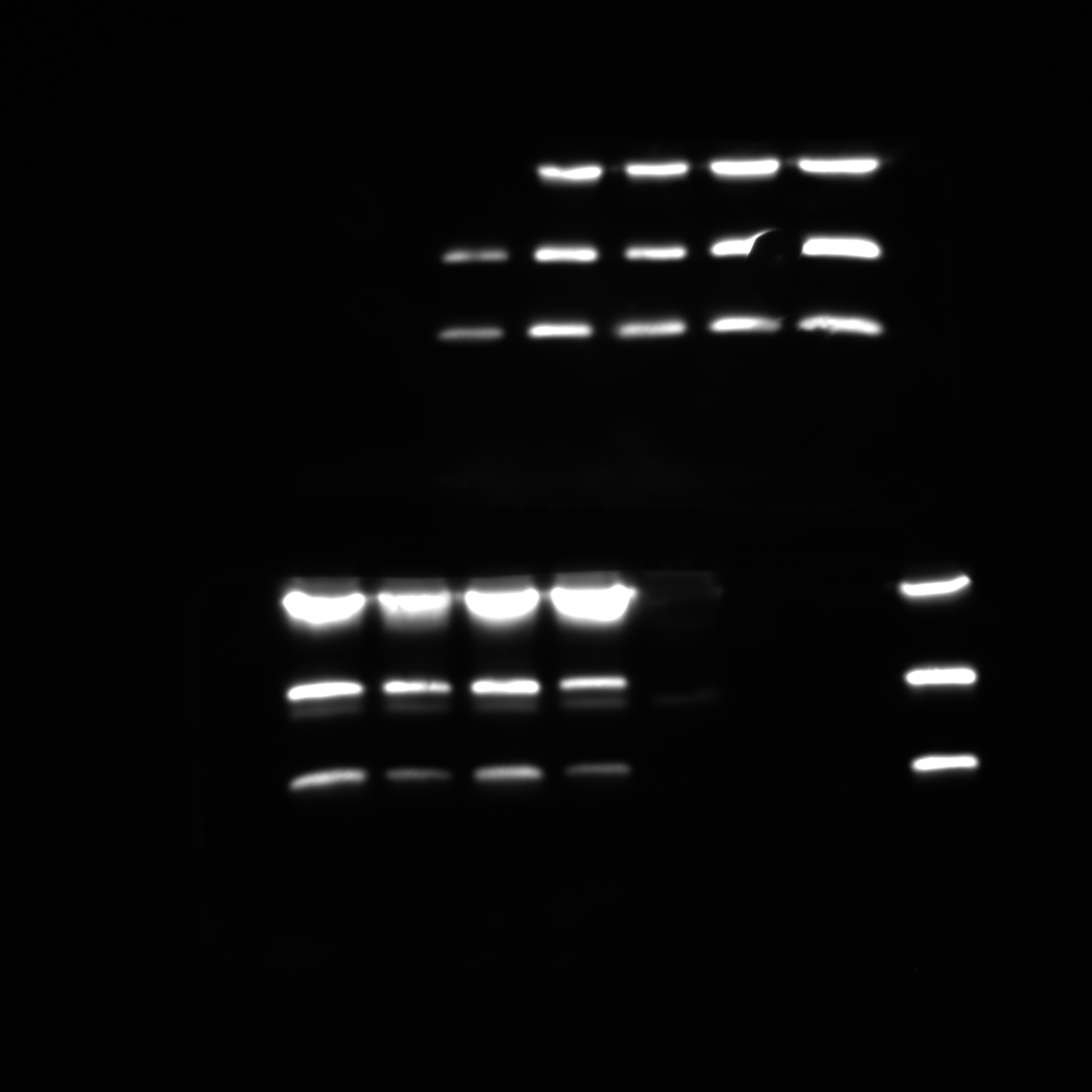

Supplement: Figure 4—source data 2. [file elife-90775-fig4-data2.zip › Figure 4-Data Source 2/Figure 4D_bottom_whole_blot.Tif]

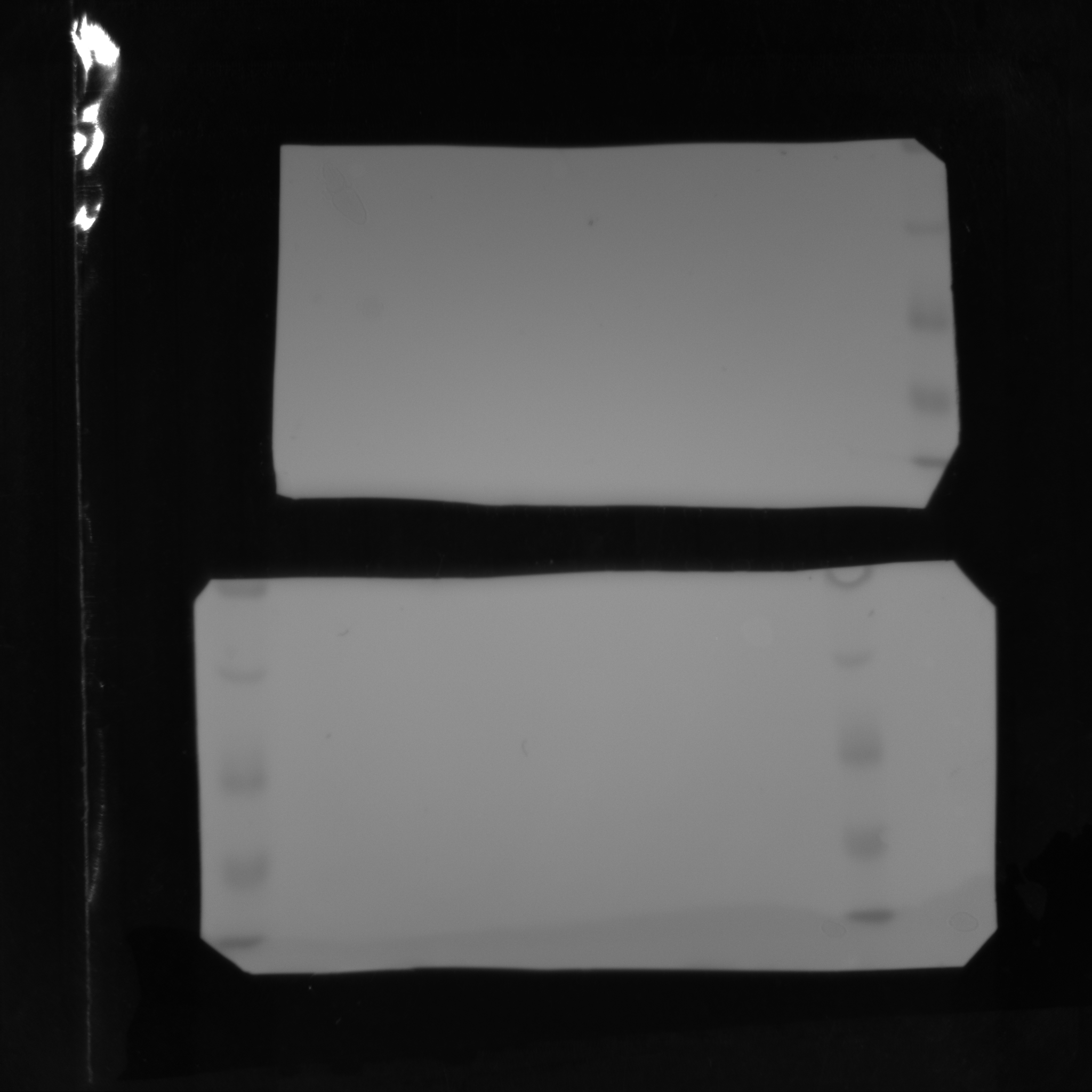

Supplement: Figure 4—source data 2. [file elife-90775-fig4-data2.zip › Figure 4-Data Source 2/Figure 4D_top_marker_precisionplusKaleidoscope.Tif]

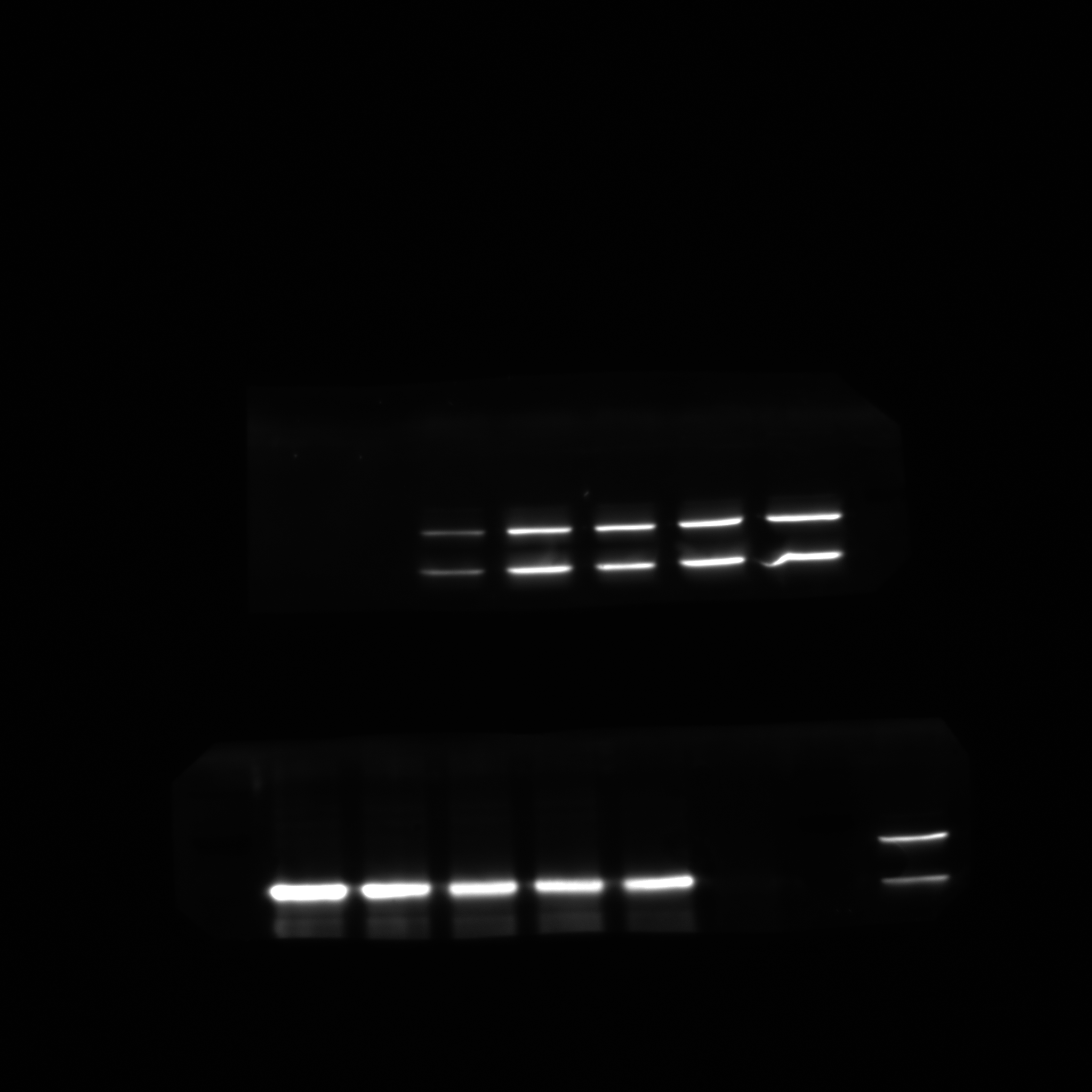

Supplement: Figure 4—source data 2. [file elife-90775-fig4-data2.zip › Figure 4-Data Source 2/Figure 4D_top_whole_blot.Tif]

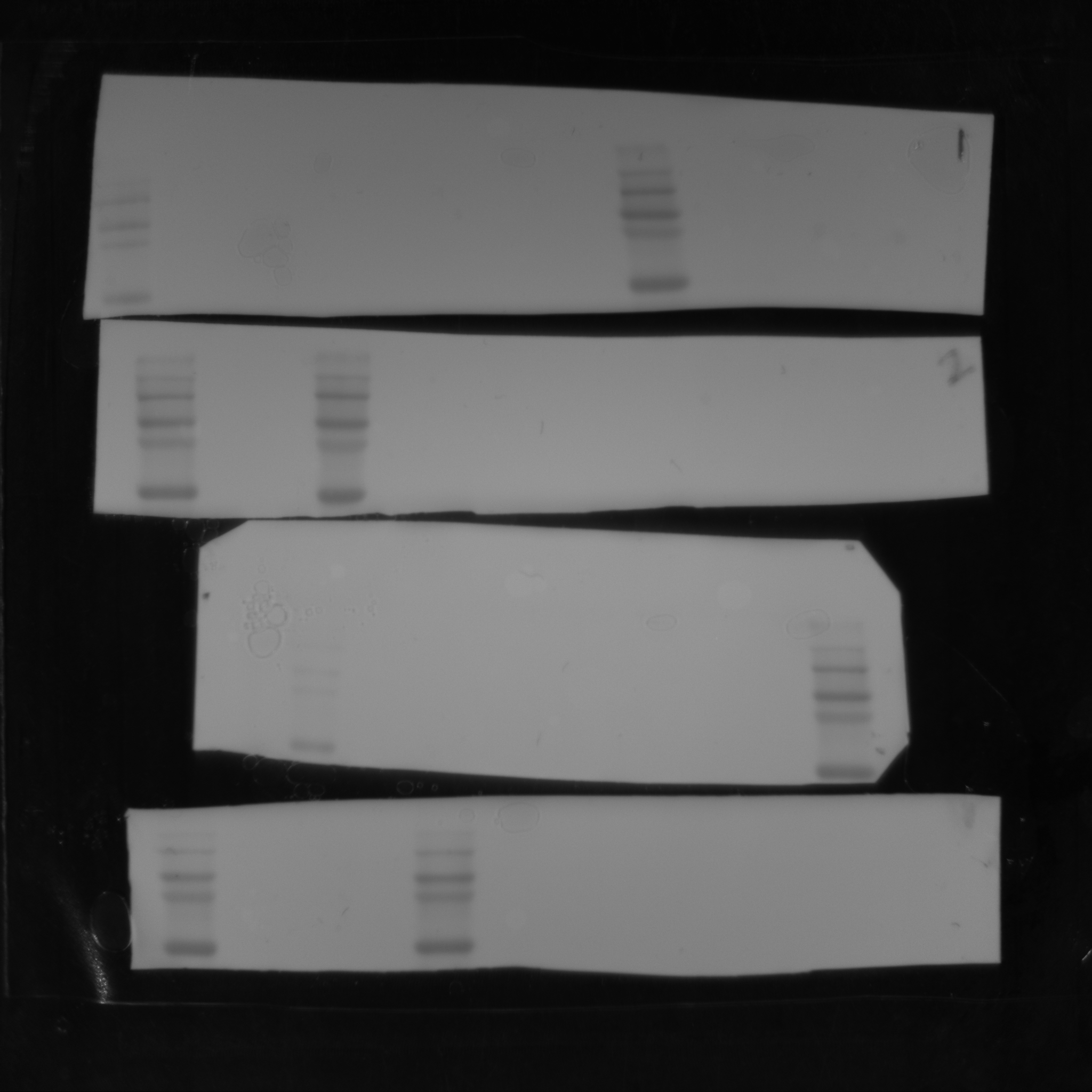

Supplement: Figure 5—source data 2. [file elife-90775-fig5-data2.zip › Figure 5-Data Source 2/Figure 5C_bTubIII(3rdblot)_marker_precisionkaleidoscope.Tif]

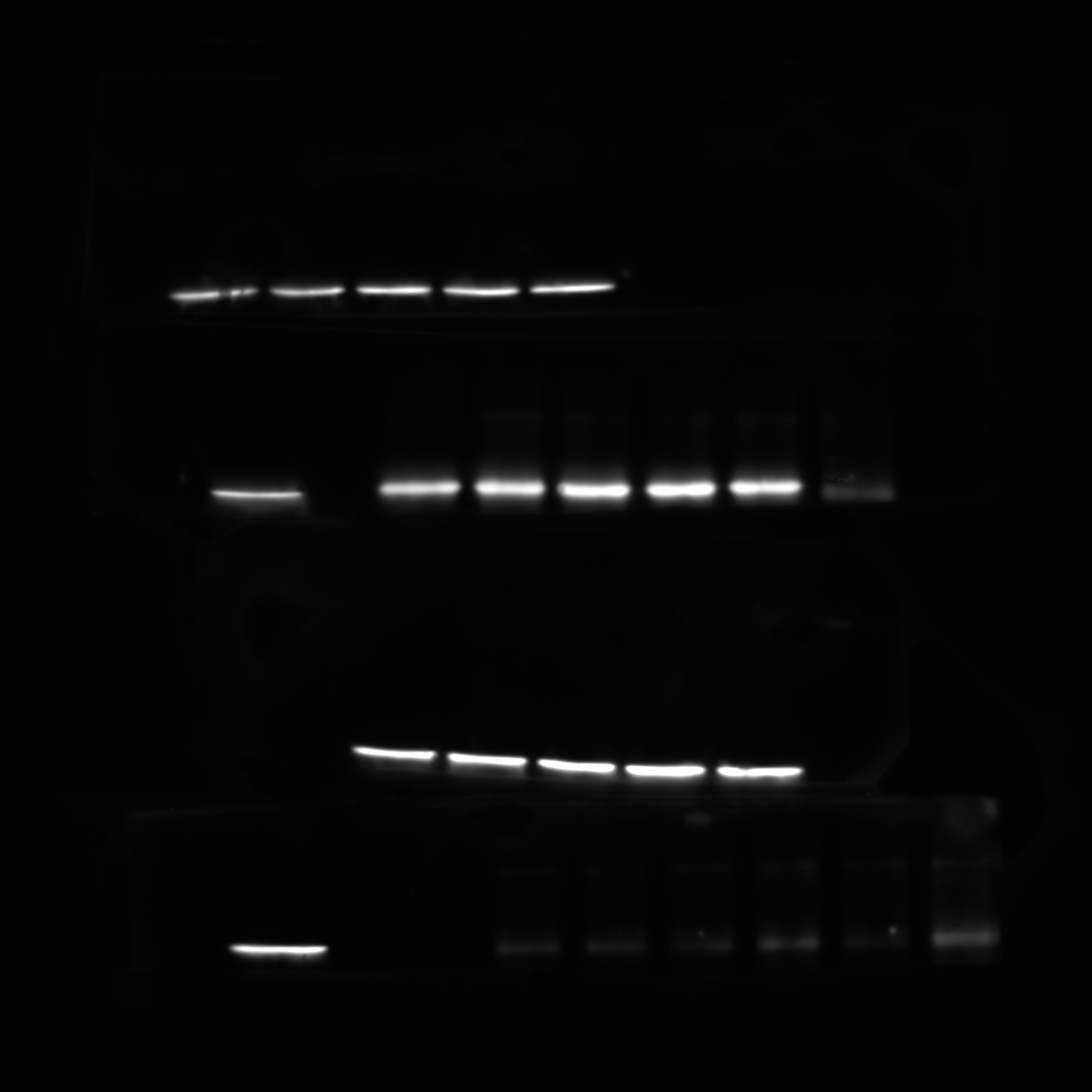

Supplement: Figure 5—source data 2. [file elife-90775-fig5-data2.zip › Figure 5-Data Source 2/Figure 5C_bTubIII(3rdblot)_whole_blot.Tif]

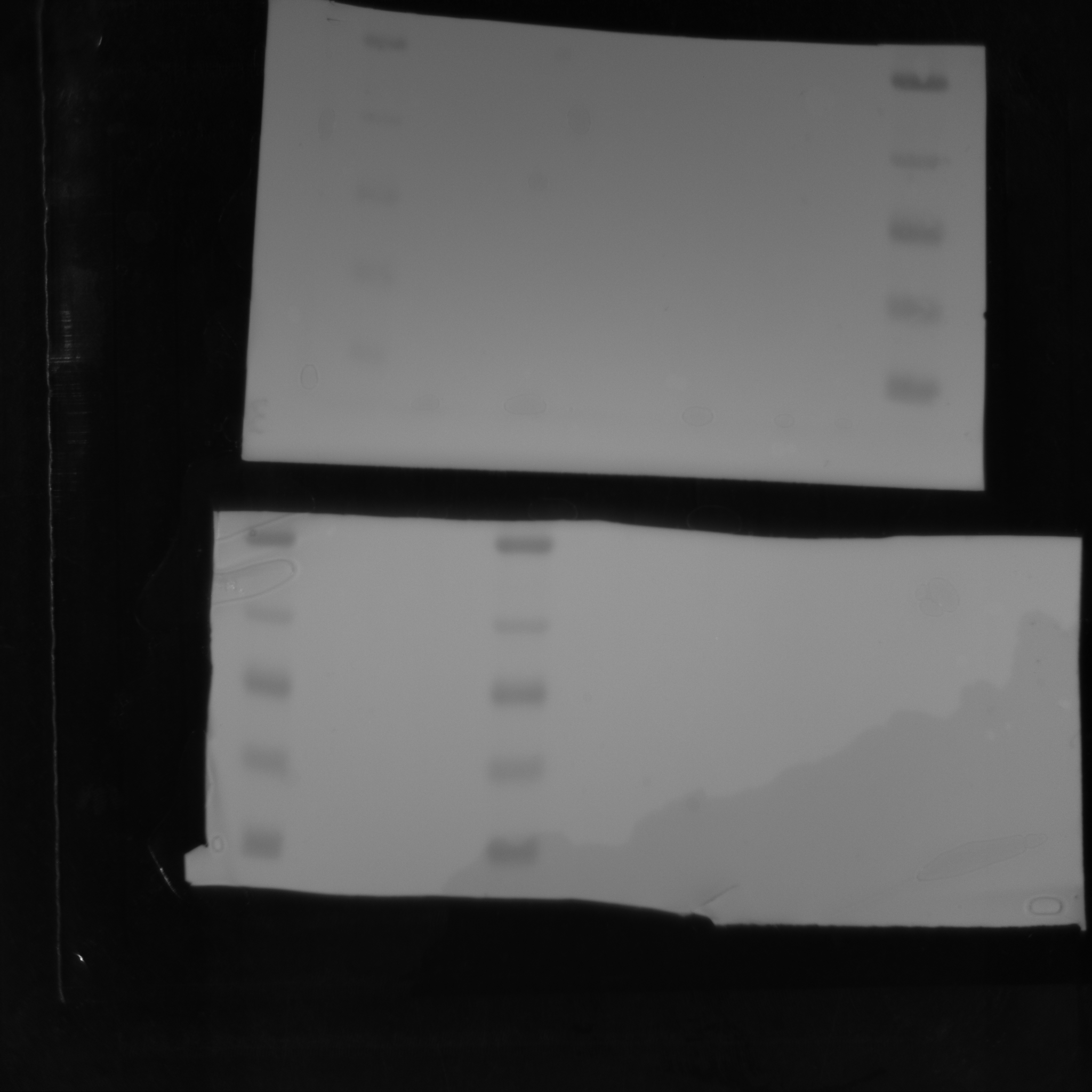

Supplement: Figure 5—source data 2. [file elife-90775-fig5-data2.zip › Figure 5-Data Source 2/Figure 5C_STX2(top)_blot_precisionkaleidoscope.Tif]

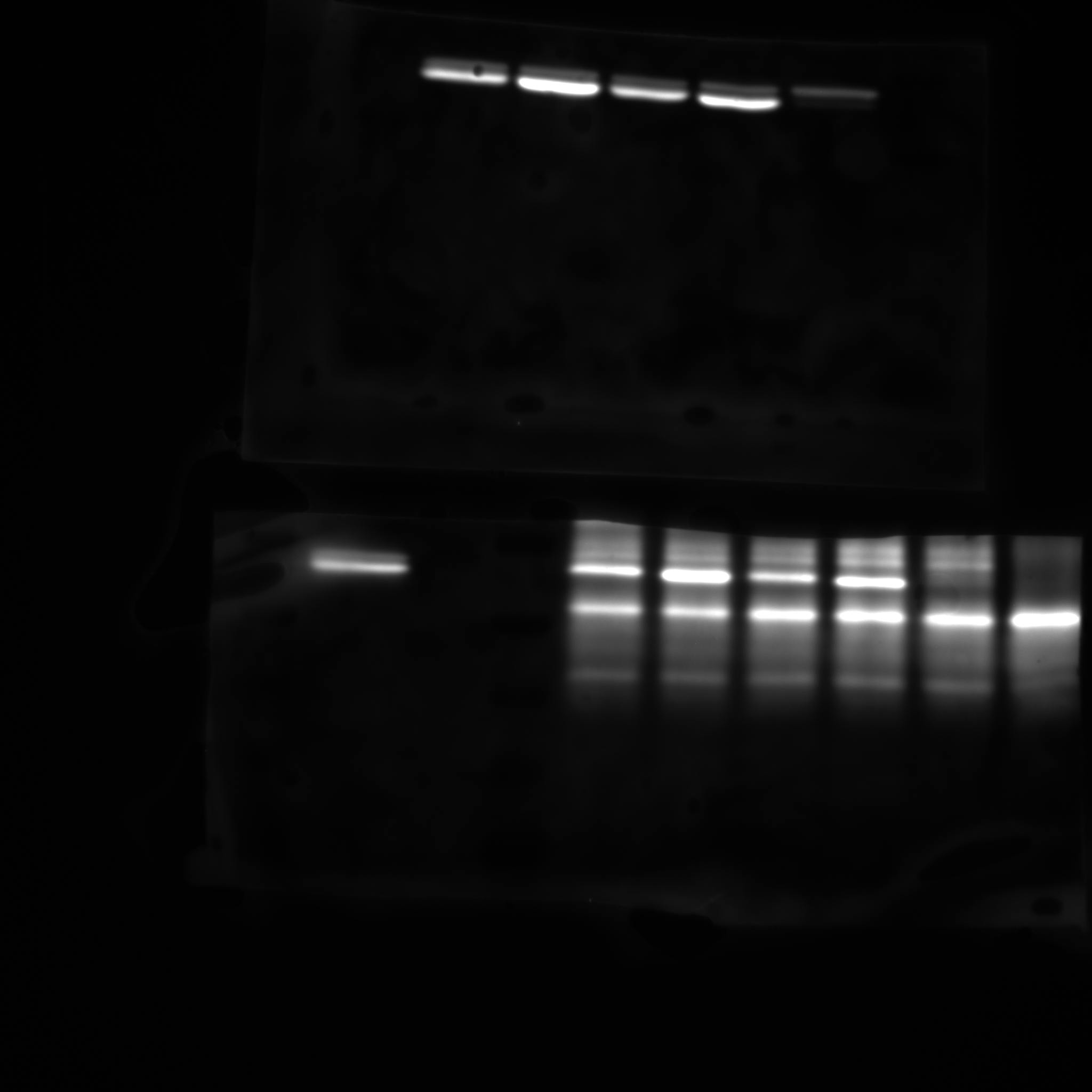

Supplement: Figure 5—source data 2. [file elife-90775-fig5-data2.zip › Figure 5-Data Source 2/Figure 5C_STX2(top)_whole_blot.Tif]

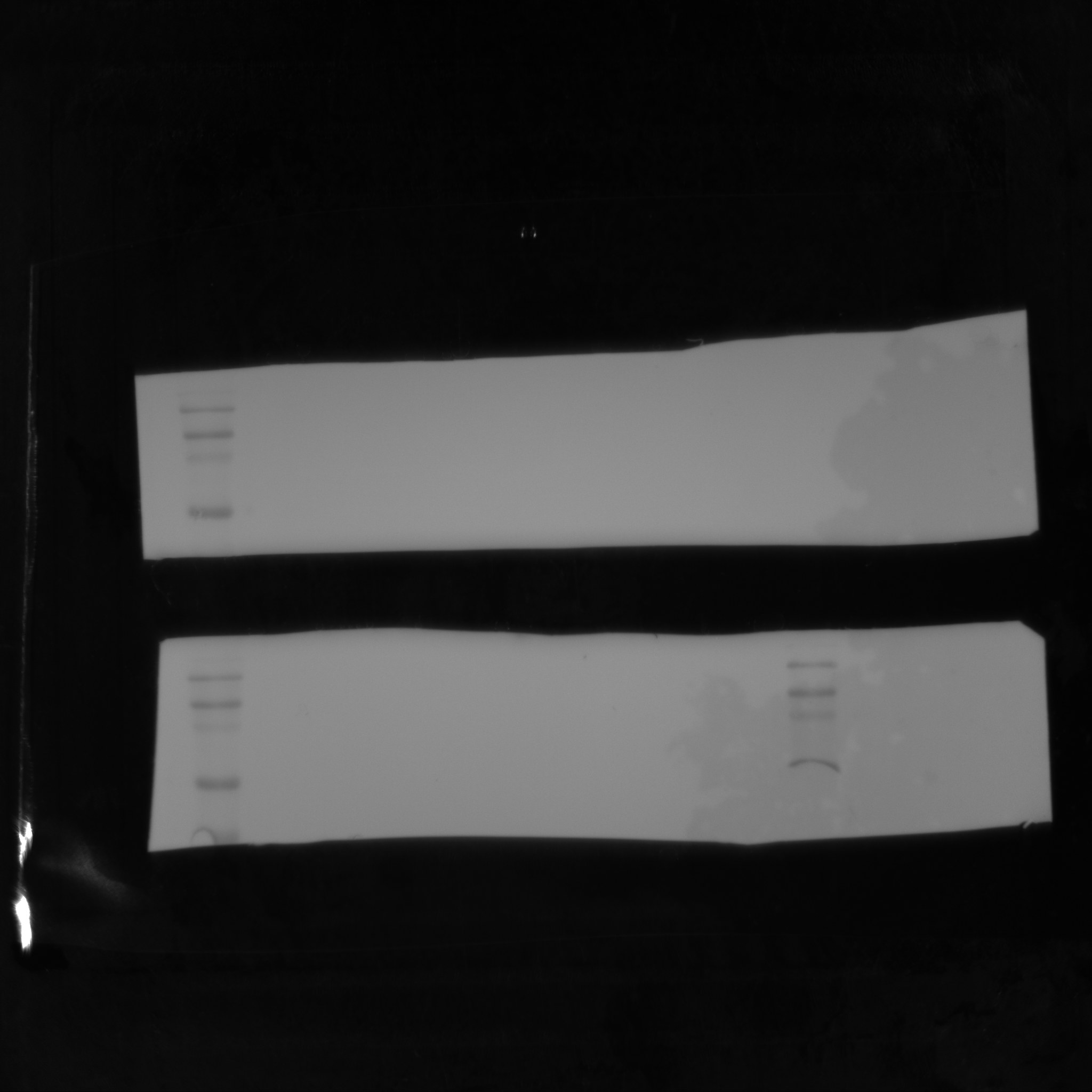

Supplement: Figure 5—source data 2. [file elife-90775-fig5-data2.zip › Figure 5-Data Source 2/Figure 5D_top_marker-precisionkaleidoscope.Tif]

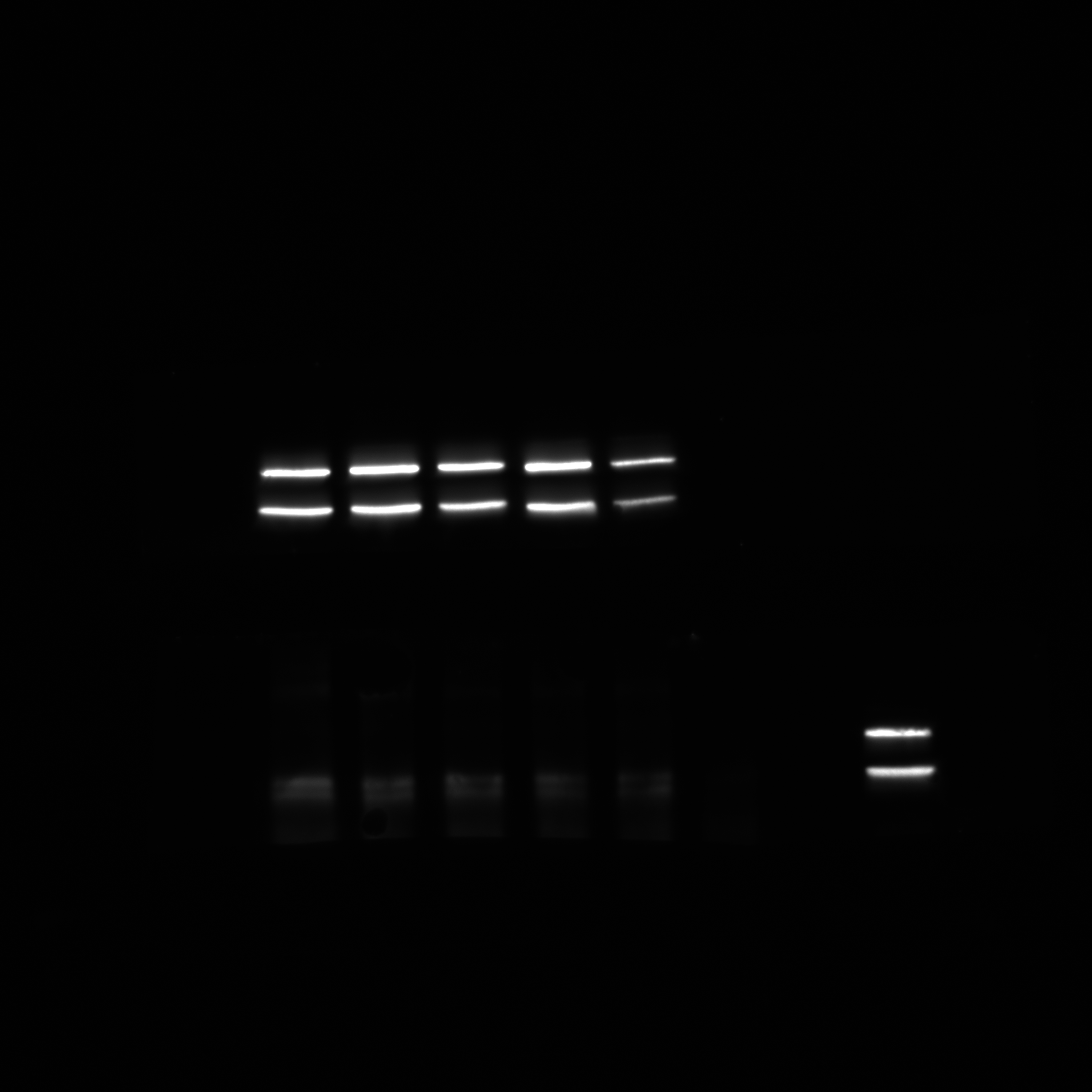

Supplement: Figure 5—source data 2. [file elife-90775-fig5-data2.zip › Figure 5-Data Source 2/Figure 5D_top_whole_blot.Tif]
