# Supplementary material for: The stability of the primed pool of synaptic vesicles and the clamping of spontaneous neurotransmitter release rely on the integrity of the C-terminal half of the SNARE domain of syntaxin-1A
Source: eLife. 2024 Mar 21;12:RP90775. doi: 10.7554/eLife.90775 (PMC10957171; doi:10.7554/eLife.90775)
Supplement: Figure 4—source data 2. [file elife-90775-fig4-data2.zip › Figure 4-Data Source 2/Figure 4 D-Data Source 2.pdf]

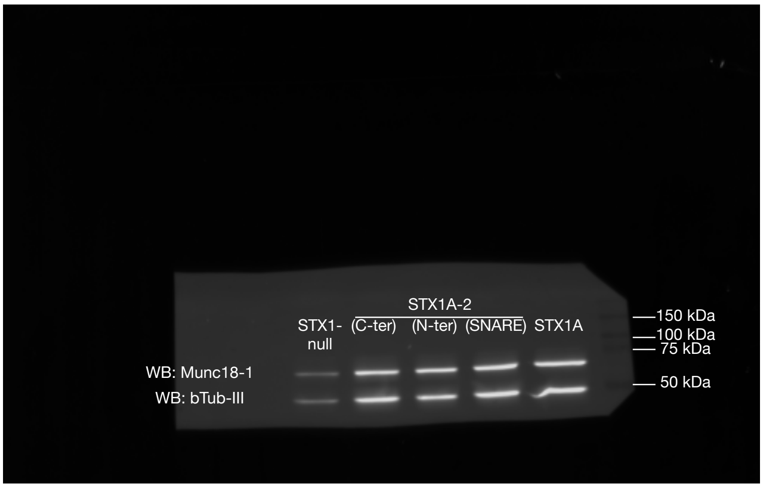

Neuronal lysates, blot **Figure 4D**. Superposition of detected protein and the image of the marker, Blot's opacity was reduced 30% for the visualization of the marker
In Figue 4. the blot was flipped horizontally for visualizetion purposes

Blot is cut between 50kDa and 37kDa for detection purposes

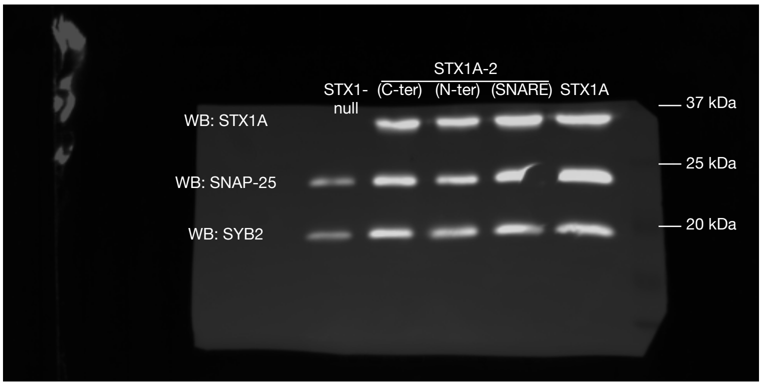

Neuronal lysates, blot **Figure 4D**. Superposition of detected protein and the image of the marker, Blot's opacity was reduced 30% for the visualization of the marker
In Figue 4. the blot was flipped horizontally for visualizetion purposes
