# Supplementary material for: The stability of the primed pool of synaptic vesicles and the clamping of spontaneous neurotransmitter release rely on the integrity of the C-terminal half of the SNARE domain of syntaxin-1A
Source: eLife. 2024 Mar 21;12:RP90775. doi: 10.7554/eLife.90775 (PMC10957171; doi:10.7554/eLife.90775)
Supplement: Figure 5—source data 2. [file elife-90775-fig5-data2.zip › Figure 5-Data Source 2/Figure 5C and D - source data.pdf]

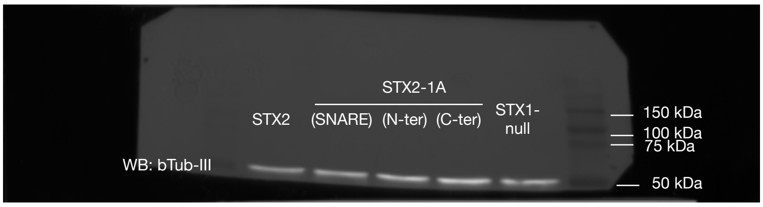

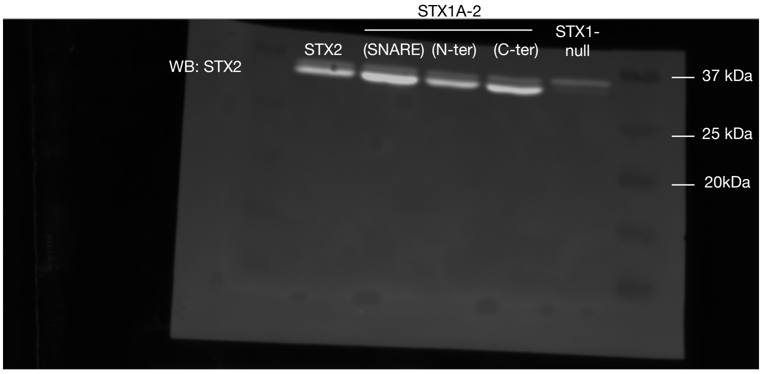

Neuronal lysates, blot **Figure 5C**. Superposition of detected protein and the image of the marker, Blot's opacity was reduced 30% for the visualization of the marker
Blots were cut between 50 and 37kDa for detection purposes

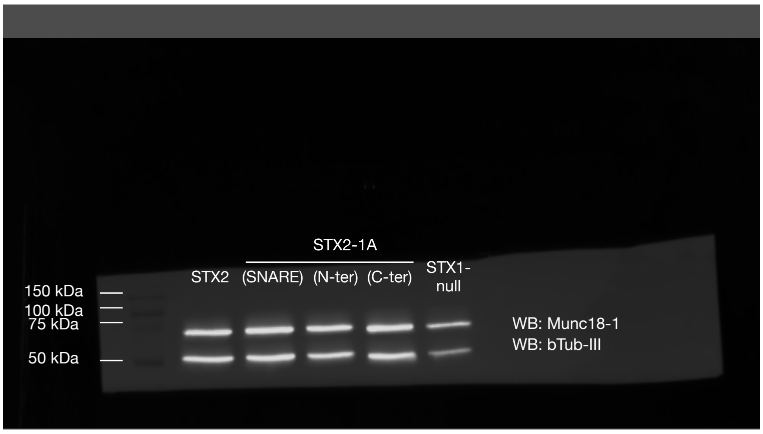

Neuronal lysates, blot **Figure 5D**. Superposition of detected protein and the image of the marker, Blot's opacity was reduced 30% for the visualization of the marker
